# Moving Kindergartens: Protocol for a 10-week feasibility study

**DOI:** 10.1371/journal.pone.0354826

**Published:** 2026-08-10

**Authors:** Elisabeth Bandak, Lise Sohl Jeppesen, Mathilde Sederberg, Charlotte Skau Pawlowski, Anna Bugge

**Affiliations:** 1 Department of Midwifery, Physiotherapy, Occupational Therapy and Psychomotor Therapy, University College Copenhagen, Copenhagen, Denmark; 2 Centre for Better Childhoods, University College Copenhagen, Copenhagen, Denmark; 3 Active Living Research Unit, Department of Sports Science and Clinical Biomechanics, University of Southern Denmark, Odense, Denmark; Karolinska Institutet, SWEDEN

## Abstract

**Objective:**

The primary aim of this study is to evaluate the feasibility of a proposed intervention designed to support the daily movement practices and fostering joy of movement in Danish kindergartens, as well as the implementation strategies intended to facilitate adoption. Additionally, the feasibility of the planned measurement and procedures for a future effectiveness study will be examined.

**Methods:**

The study uses a mixed-methods design to examine the feasibility of the intervention and the evaluation design. The planned duration of the intervention period is 10 weeks. The intervention consists of multiple integrated components co-designed with pedagogical staff, leaders, experts in kindergarten movement culture, and a team of researchers, to collectively support the daily movement practice in kindergartens. Alongside the intervention components, tailored strategies are developed to facilitate adoption and implementation. The intervention elements are categorized into four main types: 1) Formal establishment of the project; 2) Components of physical activity integrated in existing structures in the kindergarten; 3) Organizational and staff training; 4) Inspirational materials, tools and exemplary plans.

**Discussion:**

The use of a co-design approach in close collaboration between kindergartens, experts, and a team of researchers will ensure contextual relevance and shared ownership. This approach is expected to enhance acceptability, feasibility, and sustainability of the intervention. The mixed-methods evaluation design, use of theoretical implementation frameworks, and tailored strategies further strengthen the study. The findings from this feasibility study will inform the refinement of the final intervention and guide the planning of a forthcoming effectiveness study.

## Introduction

Parents, pedagogical staff, leaders, and the society in general share a vested interest in providing all children with the best possible conditions for a healthy and happy childhood, and on the long term, creating mentally, and physically healthy and resilient citizens. Research shows that physical activity and active play are important for child development, enhancing physical health, learning and well-being [1,2]. Emerging evidence demonstrates associations between physical activity and multiple health indicators already in young children such as psychosocial, cardiometabolic, bone and skeletal health [3–5], and research shows that a high level of physical activity early in life increases the chances of being physically active later in life [6–8]. Hence, interventions aiming at improving opportunities for joy of movements and integrating physical activity and active play as a natural part of all children’s lives from an early age may have an important impact on population health and well-being, both in the short and long term.

However, as in many Western countries, a large proportion of Danish children and adolescents do not meet the physical activity recommendations [9]. There is generally limited evidence on physical activity levels among pre-school aged children; however, one report found that 22% of 5-year-old children did not meet the recommendations of 60 minutes of daily physical activity [10]. Moreover, substantial differences in activity levels were observed both between the most and least active children and between boys and girls within this age group [11–14].

The Danish kindergarten setting represents an important arena for promoting children’s physical activity, active play, and movement skill development [15–17]. A Danish kindergarten is a pedagogical early childhood setting for children aged 3–6 years. Denmark has one of the most comprehensive childcare systems in the world, with about 98% of all children under the age of seven attending kindergarten [18], where they spend an average of 7.5 hours per day [19]. All Danish kindergartens are required to operate under the framework of a mandatory curriculum emphasizing play, well-being, learning, and development [20]. This provides an unparalleled opportunity for professionals to develop a daily environment in kindergartens that promotes active play, movement, and motor skills. Yet, there is evidence that only one in four kindergartens have a focus on enhancing activities that develop joy of movement and movement competences in children [21]. Furthermore, research indicate that kindergarten personnel often have limited pedagogical content knowledge related to promoting physical activity and movement in practice [22]. Additionally, the whole day care sector is experiencing significant challenges in attracting educated staff. This is reflected by a notable rise in failed recruitment attempts, from 16% to 29% for pedagogues between 2019 and 2022, and a persistent reliance on uneducated personnel, with over one-third of childcare staff lacking formal pedagogical training. Recruitment difficulties are especially pronounced in urban municipalities, where 64% of kindergarten leaders report substantial challenges in hiring qualified staff [23].

Therefore, interventions aiming at creating environments for active play and movement, must take into account both sector-wise challenges and the challenges specific to individual kindergartens. Earlier studies of physical activity interventions in children aged 3–6 years have demonstrated none to moderate effects on physical activity level, with the most effective interventions being short-termed and delivered by external experts [24,25]. However, a few more comprehensive kindergarten studies with long-term interventions delivered by trained kindergarten staff have been performed [26–28]. Despite the rigour of these studies, results have been small to non-existing both regarding changes in the children’s physical activity levels and their fundamental movement skills [29–31]. Accordingly, there is a need to investigate how interventions aiming at improving children’s physical activity levels and movement skills in the kindergarten setting can be made more effective, while remaining context-sensitive and flexible enough to accommodate the complexity of everyday practice. Such interventions should be co-designed in close collaboration with kindergarten personnel and grounded in shared responsibility and collective ownership [32].

**Fig 1:**
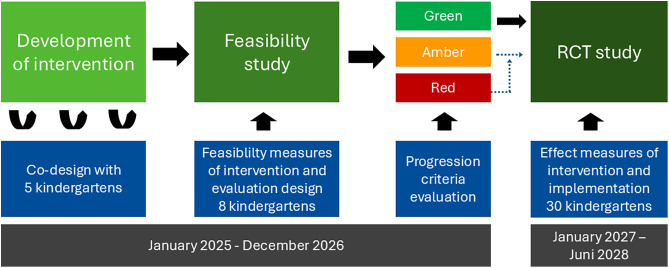
Phases and timeline for the project Moving Kindergartens.

Therefore, to support daily movement practices in kindergartens, the Moving Kindergartens project was initiated. The overall goal of the project is to enhance the kindergartens’ daily movement practices and fostering joy of movement and thereby affect children’s physical activity levels and movement competence development. The project is guided by The Medical Research Council (MRC) guidance: Framework for developing and evaluating complex interventions [33]. Hence, the Moving Kindergarten project consists of the following phases: 1) Development of an intervention, 2) Investigation of the feasibility of the intervention design and evaluation design, and if the feasibility study is successful according to predefined progression criteria: 3) Test the intervention’s effectiveness in a large-scale, cluster Randomized Controlled Trial (RCT) [34] (Fig 1).

### Aims and objectives of the feasibility study

The primary aim of this study is to evaluate the feasibility of a proposed intervention designed to support the daily movement practices and fostering joy of movement in Danish kindergartens, as well as the implementation strategies intended to facilitate adoption. Additionally, the feasibility of the planned measurement and procedures for a future effectiveness study will be examined.

Therefore, this feasibility study aims to answer the following research questions):

1. What is the level of acceptability of participation in the intervention and its content among kindergarten staff?
2. To what extent is it feasible to recruit kindergartens?
3. To what extent is it feasible to obtain child participation consent?
4. How feasible are the methods and procedures for data collection among children, staff and parents?
5. To what extent is the intervention and its implementation strategies (registered as primary outcome at clinicaltrials.gov) feasible in practice?
6. How appropriate is the intervention for the kindergarten context?
7. To what extent is the intervention adopted by kindergarten leaders and pedagogical staff, and which local adjustments are made?
8. To what extent is the intervention delivered with fidelity to its original description (adherence)?

## Methods

### Design and setting

**Fig 2:**
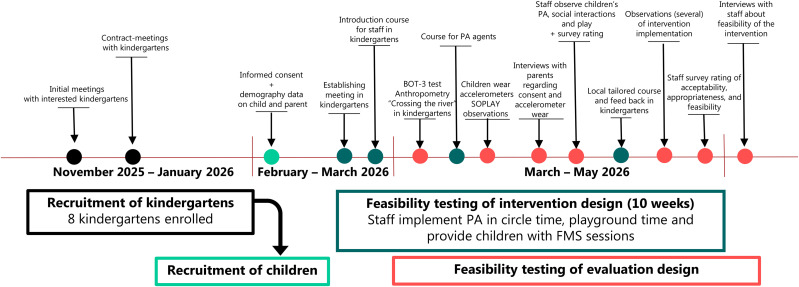
Timeline for the feasibility study.

The study uses a mixed methods design to examine the feasibility of the intervention and the evaluation design. The intervention period is 10 weeks (Fig 2). The Standard Protocol Items: Recommendations for Interventional Trials (SPIRIT) guidelines [35] has been adapted for reporting of the present study protocol as recommended [36] (S1 Table). The intervention will be delivered across 10 participating Danish kindergartens and workshops for kindergarten staff will be delivered on-site at the kindergartens and at centrally located venues.

### Participants and sample size

As this is a feasibility study, no formal sample size calculation has been performed. To ensure diversity in kindergarten size and demographic characteristics, the study aims to include 10 kindergartens representing urban and suburban areas with varied socio-economic uptake areas. Each kindergarten is expected to have between 25 and 100 children, 3–20 pedagogical staff members, and one kindergarten leader. This yields an estimated total of approximately 450–650 children aged 3–6 years, 80–120 pedagogical staff members, and 10 kindergarten leaders. Two-three kindergartens will be selected for targeted feasibility testing of the planned measurements.

### Recruitment

The 10 kindergartens will be recruited from the region of Zealand, Denmark. Kindergartens will be recruited from November 15, 2025, to January 31, 2026. The recruitment strategy includes announcements on relevant SoMe platforms (e.g., LinkedIn and Facebook), practice-oriented journals, daily news platforms, and via early childhood education consultants and other relevant contacts across several local municipalities. Interested kindergartens will receive information about participation in the feasibility study orally and written. Before participation, a written mutual contract will be signed at kindergarten level. Once a kindergarten agrees to participate, the entire kindergarten becomes part of the project; however, measurements and qualitative interviews will only be conducted with staff who provide written individual informed consent.

Written parental consent for children’s participation in the feasibility study including individual assessments will be obtained prior to study participation. Research staff will attend parent meetings or be present in kindergartens during parent drop-off and pick-up times to inform about the study and distribute the consent form. Also, information about the study and the consent form will be distributed through the kindergarten communication system reaching parents (app and online).

### Intervention

#### Intervention development phase.

The intervention was developed between January and December 2025 through a co-designing process involving stakeholders from five kindergartens (pedagogues, assistants, and kindergarten leaders), experts in kindergarten movement culture, and a team of researchers. A Design-Based Research methodology structured the process through iterative cycles of problem analysis, design, evaluation, and refinement [37–39]. The design process followed a four-phase model: Context, Workshops, Design Experiments, and Reflection providing a systematic framework for the iterative development process [40,41]. Rather than following a strictly linear sequence, the four phases unfolded in a circular and overlapping manner, allowing for flexible transitions between them.

#### Context phase.

The aim was to map opportunities, needs, and barriers related to movement practices in kindergartens. Knowledge was obtained from prior research [28,29,41,42] and through semi-structured individual interviews with pedagogues and assistants, individual interviews with kindergarten leaders, and informal interviews with experts in kindergarten culture such as municipal officials and movement consultants.

#### Workshop and Design Experiment phases.

In the first workshop, pedagogues, assistants, and leaders from the participating kindergartens (n = 19) and researchers (n = 2) co-developed the preliminary intervention framework, which was subsequently tested over five months during the first Design Experiment phase. In a second workshop, the intervention framework was refined based on insights from the Design Experiment phase. Thereafter, the revised version of the intervention framework was tested over two months in a second Design Experiment phase in two of the five kindergartens. Data was collected through field observations and informal interviews in kindergartens.

#### Reflection phase.

Insights from the development process were supplemented by final interviews with pedagogues and leaders from the participating kindergartens, focusing on their reflections on the intervention’s potential and challenges in practical implementation. In addition, continuous reflection meetings were conducted throughout the development phase at several levels including staff from the kindergartens, experts in kindergarten movement culture, and researchers. These dialogues served as key forums for collective reflection, co-designing, and adjustment of both research and design processes, enriching the understanding of the intervention’s development and implementation potential.

#### Intervention feasibility phase.

Building on the intervention designed during the development phase, the intervention in the feasibility study spans 10 weeks from March 2, 2026, to May 8, 2026, whereas in the full-scale effectiveness study, the intervention will extend over one year. The intervention developed in the first phase of this study (Intervention development phase) consists of multiple integrated components designed to collectively support the daily movement practice in kindergartens. Alongside the intervention components, tailored strategies are developed to facilitate adoption and implementation, and these are classified using the ERIC compilation adapted for early childhood education and care (ECEC) settings [43,44].

The intervention elements can be categorized into four main types (described in detail in Table 1):

**Table 1:** Intervention elements.

Intervention elements	Actors	Description of strategies for implementation	*ERIC/SISTER implementation strategy
Formal establishment of the project	Research team and kindergarten staff	Formalized contract stating the responsibilities of both parties (kindergartens and research team)	Obtain formal commitments (Improve implementers’ buy-In), (No 51)
	Research team through the kindergarten	Information about the project (verbal and written) and the informed consent procedure targeted parents. This is delivered from the research personnel to the kindergarten staff, which informs the parents.	Communicate with students, families, and other staff to enhance uptake and fidelity (No 56) Prepare families and students to be active participants (No 58)
Components of physical activity integrated into existing structures in the kindergarten	Kindergarten staff	Physical activity and active play implemented in daily child-group “gatherings”. The target is 25 minutes four times a week.	Develop a detailed implementation plan or blueprint (No 5)
	Kindergarten staff	Physical activity and active play facilitated in “playground time”. The target is 3 x15 minutes four times a week.	Develop a detailed implementation plan or blueprint (No 5)
	Kindergarten staff (2–3 from each kindergarten)	Movement skill training integrated into active play 30 minutes once a week.	Develop a detailed implementation plan or blueprint (No 5)
	Kindergarten staff	Physical activity and active play on the agenda at staff meetings. Research team delivers the meeting agendas. Meetings are held approximately every 3^rd^ week.	Develop a detailed implementation plan or blueprint (No 5) Organize kindergarten staff implementation team meetings (No 32) Conduct local consensus discussions. Create a professional learning collaborative (No 40)
	Kindergarten staff (2–3 from each kindergarten)	Establishing movement agents (champions) to ensure a focus on physical activity in each kindergarten	Identify and prepare champions (No 26) Make training dynamic (No 43) Shadow other experts (No 45)
Organizational and staff training	Expert educators	Courses and training in the components of physical activity for pedagogical staff	Conduct ongoing training (No 39) Make training dynamic (No 43) Work with educational institutions (No 47)
	Expert educators	Courses for kindergarten leaders about relationship between children’s physical activity, wellbeing, and learning	Recruit, designate, and train for leadership (No 34)
	Expert educators	Three on-site visits in each kindergarten providing feedback, supervision and support	Provide ongoing consultation/coaching Conduct educational outreach visits (No 38) Provide practice-specific supervision (No 14) Make training dynamic (No 43)
Inspirational materials, tools and exemplary plans	Research team	”Lesson plans” for “gatherings” Catalogue of activities for the playground Didactic tool for planning activities Movement skills training program (playful activities) Meeting agendas and process tools	Develop and distribute educational materials (No 41 and 43)

**Table legend**: Numbers (No) listed in the ERIC/SISTER column refer to strategies defined in Table 2 in [43].

1) Formal establishment of the project
2) Components of physical activity integrated in existing structures in the kindergarten
3) Organizational and staff training
4) Inspirational materials, tools and exemplary plans

The intervention elements and implementation strategies described in Table 1 represent the level of practice that participating kindergartens are expected to strive towards throughout the intervention period. In this setup, children will be exposed to multiple daily opportunities for physical activity and active play — including child group gatherings integrating movement, play, storytelling and language development, structured active play sessions during outdoor periods, and a weekly session led by the movement agent focusing on fundamental movement skills. Together, these components form a meaningful activity dose that, based on existing evidence, is expected to elicit measurable effects on children’s physical activity and movement competences. Importantly, a key objective of the feasibility study is to examine whether such a level of implementation is in fact feasible within the everyday realities and routines of kindergarten practice.

### Outcomes and data collection

Data collection will take place throughout the 10 weeks of the feasibility study (from March 2, 2026, to May 8, 2026). Each data collection method is described below and linked to the corresponding outcome measure as outlined in Table 2.

**Table 2:** Outcomes in the moving kindergartens feasibility study.

Outcomes	Outcome definition	Target group	Methods
Acceptability	The overall perception of whether the project is found desirable to participate in	Leaders and pedagogical staff	Semi-structured group Interviews (pedagogical staff) Individual interviews (leaders)
	The extent to which the intervention is found acceptable	Leaders and pedagogical staff	Survey (AIM)
	The likelihood of providing informed consent for child participation in the project	Parents	Parental survey Semi-structured telephone interviews
Feasibility	The extent to which the recruitment strategy leads to inclusion of sufficient kindergartens	Leaders	Recruitment data registration Semi-structured interviews
	The feasibility of the process of obtaining child participating consent and demography and background information of the child and parents	Parents	Recruitment data registration Parental survey Semi-structured telephone interviews
	The feasibility of data collection on child level: physical activity, fundamental movement skills, social interaction, sleep duration, leisure time activities, and anthropometrics	Pedagogical staff Parents Trained test personnel	Participatory field observations, Semi-structured group interviews (pedagogical staff) Semi-structured individual interviews (parents and trained test personnel)
	The feasibility of data collection and procedures targeting leaders and pedagogical staff	Leaders and pedagogical staff	Semi-structured group interviews (pedagogical staff) Individual interviews (leaders) Survey (custom-made) Data registration
	The extent to which the intervention is found to be understandable and feasible	Leaders and pedagogical staff	Semi-structured group interviews (pedagogical staff) Individual interviews (leaders) Participatory field observations Survey (FIM) (pedagogical staff and leaders)
Appropriateness	The relevance and fit of the intervention in relation to everyday routines, competences, priorities, values, and structural requirements of the kindergarten	Leaders and pedagogical staff	Semi-structured group interviews (pedagogical staff) Individual interviews (leaders) Participatory field observations Survey (IAM) (pedagogical staff and leaders)
Adoption	How the proposed intervention is adopted, and eventually adjusted	Leaders and pedagogical staff	Semi-structured group interviews (pedagogical staff) Individual interviews (leaders) Participatory field observations Implementation log (poster with daily check boxes) Survey (pedagogical staff)
Fidelity	The extent to which meetings and educational activities are completed	Leaders and pedagogical staff	Semi-structured group interviews (pedagogical staff) Individual interviews (leaders)
	The adherence to the intervention description	Leaders and pedagogical staff	Implementation log (poster with daily check boxes) Participatory field observations

**Table legend**: Abbreviations: AIM: Acceptability of Intervention Measure, IAM: Intervention Appropriateness Measure, and FIM: Feasibility of Intervention Measure.

### Interviews

Interviews will be conducted and recorded with different informants (parents and kindergarten staff/leaders) and follow specific interview guides reflecting the aims and outcomes of the interviews.

#### Interviews with pedagogical staff and leaders.

Semi-structured group interviews will be conducted with pedagogical staff (3–5 participants) on-site in each of the 10 kindergartens during the final weeks of the intervention period. In addition, individual semi-structured interviews will be conducted with the leader of each kindergarten.

#### Interviews with parents.

Semi-structured individual telephone interviews will be conducted with 4–5 randomly selected parents after giving informed consent and after the children have worn accelerometers measuring physical activity.

### Participatory field observations

Participatory field observations will be conducted 1–2 days in all kindergartens. Furthermore, in-depth observations will be performed in two of the kindergartens over 1–2 consecutive weeks. The aim is to gain a deeper understanding of how the intervention unfolds in everyday practice over an extended period. These observations will follow a structured observation guide and investigate the outcomes described in Table 2. In short, the observations begin with broad descriptions aimed at capturing everyday practices, rhythms, paradoxes, and unforeseen events, describing participants, interactions, routines, and temporal patterns [45]. The focus is then progressively narrowed toward the core elements of the intervention through focused observations. In the remaining kindergartens, only one-day focused observations are carried out, directed specifically at the intervention’s key components (e.g., movement words in circle time, staff roles on the playground, spontaneous movement play, and logistical conditions). This differentiated, progressively narrowing design ensures both in-depth contextual understanding in the two primary sites and broader testing of the intervention elements across additional settings.

#### Surveys.

To assess the feasibility of both the intervention content and the data collection, surveys will be distributed as described below.

Acceptability of Intervention Measure, Intervention Appropriateness Measure, and Feasibility of Intervention Measure

Pedagogical staff and leaders will complete a survey including measures of acceptability, appropriateness, and feasibility of the intervention. The survey will collect data from the perspective of the pedagogical staff. It comprises 12 self-reported items derived from three validated constructs—Acceptability of Intervention Measure (AIM), Feasibility of Intervention Measure (FIM), and Intervention Appropriateness Measure (IAM) [46], which collectively assess the domains of acceptability, feasibility, and appropriateness. Each item is rated on a 5-point ordinal scale (1 = Completely disagree, 2 = Disagree, 3=Neither agree nor disagree, 4 = Agree, and 5 = Completely agree). The survey will be translated and adapted to the cultural and linguistic context of Danish kindergartens. A Danish translation of the instrument was recently developed following a standardized four-step translation protocol and used in a school-based implementation study [47,48]. Our translation will follow the same four-step procedure. Additionally, custom-made questions regarding adoption of the physical activity intervention components will be included. The combined survey will be distributed both as an online survey and in a paper-version during the last weeks of the feasibility study.

#### Child Movement Engagement and Social Participation.

Children’s general movement engagement and social participation in physical activity and active play will be assessed using a staff-rated questionnaire. It consists of 10 items; eight items from MoveEarly questionnaire regarding child involvement and well-being (Aadland 2025 personal communication), and two custom-made items rated on a 5-point Likert scale (Never to Very often). The questionnaire must be completed by the staff members most familiar with the child, based on their daily interactions and experiences. The questionnaire will be distributed in the last weeks of the feasibility study both as an online survey and a paper-version.

#### Parental survey.

In a parental survey, baseline information will be collected, including information on the child (status of preterm birth, numbers of siblings, transportation to and from kindergarten) child leisure time (participation in organized physical activity, recreational screentime), parents’ demography (educational level). This survey will be administered online through the kindergartens communication system and in a paper version.

#### Recruitment data and implementation log.

To assess the feasibility of the recruitment strategy and the fidelity of the intervention, data will be collected as described below.

#### Recruitment registration.

Information about the number of kindergartens approached, the number agreeing to participate, and the number of parents providing consent will be systematically recorded in administrative records. Recruitment feasibility will be evaluated by calculating response and participation rates at both institutional and individual levels. These indicators will inform assessment of recruitment success and guide adjustments for the future effectiveness study.

#### Implementation log.

Fidelity of the intervention will be monitored using an implementation log consisting of a large wall poster displayed in each participating kindergarten. The poster functions as a quantitative log, featuring daily checkboxes in the intervention period for each component of the intervention, along with an option for ‘none’. The poster covers the entire intervention. In each kindergarten, movement agents are responsible for the use of the poster. They will receive instructions on how to use the poster and will be reminded weekly via email or SMS throughout the intervention period. Data recorded on the posters will be collected physically by the research team. This implementation log will provide quantitative data on the frequency of intervention activities throughout the intervention period and indicate whether the different intervention elements – and their target implementation level – were feasible.

#### Feasibility of data collection on child level.

To address the feasibility of chosen methods and procedures for data collection on child level, the test procedures on children’s physical activity, fundamental movement skills, social interaction, and anthropometrics will be assessed in this feasibility study. The feasibility testing of child level assessments will be conducted in two-three kindergartens (approximately 100–300 children in total). To ensure children’s well-being, all measurements and activities will take place in a playful and age-appropriate environment, designed to make children feel safe and engaged. Emphasis will be placed on creating a setting characterized by familiarity, curiosity, and respect for the children’s perspectives.

#### Anthropometrics.

Children’s’ height will be measured using a stadium meter (West Sussex, UK) with a precision of 0.5 cm and body weight will be measured to one decimal (0.1 kg) using an electronic scale (Tanita WB-150 SMA, Tokyo, Japan). Children will not be allowed to see their height and weight measure.

#### Time in kindergarten.

Attendance data for each participating child will be extracted from the app-based attendance systems used in the participating kindergartens.

#### Objective measures of physical activity.

Physical activity will be objectively assessed using accelerometers (AX3, Axivity, Newcastle, UK) worn for seven consecutive days by children. The devices will be attached to the right thigh using medical tape [49]. The data collected will be analyzed to identify the number of children fulfilling the minimum wear time criteria. Furthermore, the data quality in terms of variation in activity intensity and types will be evaluated.

#### Systematic observations of physical activity and social interaction (SOPLAY).

The System for Observing Play and Leisure Activity in Youth (SOPLAY) will be used for direct observations of the children’s physical activity and social interaction at the outdoor playgrounds. SOPLAY is a reliable and validated structured observation tool used to record movement patterns in a defined area such as a schoolyard [50]. The original version of SOPLAY will be modified to fit the specific purpose of this study by including information on social interaction, also done in previous studies using SOPLAY [51]. Data will be collected following a strict procedure during playground time at three days in the same time slot and in the same two-three kindergartens measuring children’s physical activity using accelerometers. The data captured from SOPLAY will be gender, physical activity intensity (sedentary, moderate, vigorous), play activities, and social interaction. Further weather and temperature for observation days will also be registered. The time consumption and needed manpower obtaining the data and the data quality in regard to inter-rater variability will be used to address the feasibility of using SOPLAY in this kindergarten study.

#### Fundamental movement skills.

Fundamental movement skills will be assessed using a short form of the Bruininks-Oseretsky Test of Motor Proficiency, second edition (BOT-2) [52]. The short form of the BOT-2 is recommended for use in cross-sectional studies to assess overall motor proficiency in healthy children and youth aged 4–21 years [53]. The BOT-2 Short Form comprises a subset of 14 items. Nine tests of gross motor skills (including tests for balance, bilateral coordination, strength and agility), and five tests of fine motor skills (including tests for precision, integration, manual dexterity, upper-limb coordination). Assessments of fundamental movement skills will be carried out by trained test personnel. The tests will be conducted individually in a quiet room in the kindergarten, with a total duration of approximately 15 minutes per child. The data gathered will be used to address the feasibility of BOT-2 in a kindergarten context including data quality of the test results, time to complete the test, and numbers of completed tests.

### Data analysis

The collected data on acceptability, feasibility, appropriateness, adoption, and fidelity will be analyzed as follows: All interviews (on-site group, on-site individual, and telephone interviews) will be transcribed using the AI-transcription tool Good Tape (goodtape.io, Denmark) and manually adjusted for errors. All interviews undergo a thematic analysis [54], guided by the outcomes of interest in terms of feasibility of the intervention (e.g., acceptability and adoption) [55]. Additionally, given the feasibility nature of this study, data quality will be assessed descriptively, focusing on completeness, consistency, and adherence to data collection procedures, as well as time use and test completion rates on child and staff level. These indicators will be used to inform refinements to study design and outcome measures for future trials rather than to draw effectiveness conclusions.

For each of the constructs in the AIM, IAM, and FIM survey a total score for each domain will be calculated by averaging the item responses [46], while the total scores of the AIM and FIM constructs are part of the progression criteria (see below). In the custom-made staff-rated survey, all 10 items will be reported individually.

Results are expected to be generated by December 31, 2026.

### Progression criteria

To assess whether the feasibility study is successful, a set of progression criteria have been developed and will be evaluated at the end of the feasibility study (Table 3).

**Table 3:** Progression criteria.

Progression criteria	Green: Proceed to RCT Quant	Amber: Adjust before proceeding to RCT Quant	Red: Issue must be solved before proceeding to RCT Quant
**Acceptability** (parents)	≥ 65% of eligible children are included (Administrative data)	50-65% of eligible children are included (Administrative data)	< 50% of eligible children are included (Administrative data)
**Acceptability** (staff)	Pedagogical staff score mean total of ≥ 4.0 (AIM survey)	Pedagogical staff score mean total of ≥ 3.0- < 4.0 (AIM survey)	Pedagogical staff score mean total of < 3.0 (AIM survey)
**Adoption** (staff)	≥ 66% of enrolled pedagogical staff participated in courses and training activities (Administrative data)	50-66% of enrolled pedagogical staff participated in courses and training activities (Administrative data)	< 50% of enrolled pedagogical staff participated in courses and training activities (Administrative data)
**Adoption** (staff)	≥ 66% enrolled pedagogical staff report use of physical activity intervention components (survey).	50-66% of enrolled pedagogical staff report use of physical activity intervention components (survey).	< 50% of enrolled pedagogical report use of physical activity intervention components (survey).
**Feasibility** (staff)	Pedagogical staff score mean total of ≥4 (FIM survey)	Pedagogical staff score mean total of ≥3.0- < 4.0 (FIM survey)	Pedagogical staff score mean total of <3.0 (FIM survey)
**Feasiblity** (children)	≥ 65% of enrolled children from 2–3 kindergartens have worn the accelerometer (data collection)	50-65% of enrolled children from 2–3 kindergartens have worn the accelerometer (data collection)	< 50% of enrolled children from 2–3 kindergartens have worn the accelerometer (data collection)

Abbreviations: AIM: Acceptability of Intervention Measure, FIM: Feasibility of Intervention Measure

If all the progression criteria of the feasibility study are fulfilled (green), the project will proceed to the RCT phase without modifications (Fig 1). If any criteria are rated as amber, this indicates that adjustments and amendments are required based on insights gained during this study. These amendments will be applied before initiating the RCT study. If any of the progression criteria are red, this signals that substantial changes are required. In such cases, a detailed justification for the proposed modifications must be provided before proceeding to the RCT. To ensure the relevance of potential amendments these will be undertaken by the research team in close collaboration with both pedagogical staff, kindergarten leaders, and experts involved in the development phase.

### Ethics

The study is approved by Research Ethics Committee at the University of Southern Denmark (25–14029) and will be conducted in accordance with the Declaration of Helsinki. Personal information will be collected through secure, encrypted survey platforms and stored on password-protected servers. Access will be restricted to the research team only. All data will be handled in compliance with the General Data Protection Regulation (GDPR). All pedagogical staff and leaders will receive both written and verbal information about the study. They will have the opportunity to ask questions to members of the research team before providing written, informed consent. Similarly, parents or legal guardians of children in participating kindergartens will receive written information and will be offered the opportunity to clarify any questions with members of the research team before providing written, informed consent on behalf of their child. Children’s certificates will be collected for all study personnel prior to study participation. Children’s certificate (*børneattest*) is a special type of criminal-record certificate issued by the Danish Police [56]. It is legally required to be obtained by employers, associations, and public institutions when hiring or engaging people who will have direct contact with children under the age of 15.

## Discussion

This study will generate new knowledge about the potential for implementing an intervention designed to support the daily movement practices and fostering joy of movement in kindergartens. Further, the results will inform the final refinement of the intervention, as well as the strategies for implementation and the effect outcomes for the forthcoming RCT study.

Promoting childhood physical activity in a kindergarten setting is complex, as institutional structures, cultural norms, and the pedagogical staff’s personal motivation for and prior experience with physical activity all play a significant role in shaping opportunities for movement in daily practice [32,57,58].

The MRC framework emphasizes the importance of incorporating co-design in intervention development to achieve meaningful changes in real-world settings [33]. Therefore, co-designing an intervention with pedagogical staff and kindergarten leaders may enhance its acceptability and adoption, ultimately increasing children’s physical activity levels and movement competence development.

### Strengths and potential challenges of the study design

Interventions developed using co-design have been proposed as a viable strategy for addressing complex challenges, as it integrates current evidence on best practices and local knowledge to develop context-specific solutions [33]. In Moving Kindergartens, the participatory approach, characterized by co-design and close collaboration between kindergartens and the research team, is anticipated to be a key strength of this feasibility study [59]. The study’s comprehensive evaluation design, incorporating both quantitative and qualitative data collection methods, represents an additional methodological strength [60], as well as a strength is the use of relevant theoretical frameworks guiding for example the choice of feasibility outcomes [61]. Although the co-design approach may increase the likelihood of sustainability, a potential challenge of the study is the long-term engagement of pedagogical staff, which may decline over time particularly due to possible staff and leadership turnover within kindergartens. However, this feasibility study will provide valuable insights into the level and nature of support required from the research team, kindergarten leaders, and staff to maintain focus on movement practices and active play in the kindergartens. In addition, external factors may also pose challenges to implementation. For example, local or municipal economic constraints could result in reduced resources at the kindergarten level. Nevertheless, the primary focus of the study described in the present protocol is to evaluate the feasibility of the proposed intervention designed to be implemented without additional resources in terms of staff or facilities.

### Dissemination plans

The findings from this feasibility study will be synthesized and submitted for publication in peer-reviewed journals, with a preference for open-access availability. Results will also be presented at relevant national and international scientific conferences. In addition, plain language summaries will be prepared and shared with stakeholders involved in intervention development, to study participants, and to the general public. These summaries will be disseminated through social media platforms and non-peer-reviewed journals.

## Supporting information

S1 Table SPIRIT 2025 Checklist. (PDF)
